# Exploring Soil Pollution Patterns Using Self-Organizing Maps

**DOI:** 10.3390/toxics10080416

**Published:** 2022-07-25

**Authors:** Ilaria Guagliardi, Aleksander Maria Astel, Domenico Cicchella

**Affiliations:** 1 National Research Council of Italy—Institute for Agricultural and Forest Systems in Mediterranean (CNR-ISAFOM), Via Cavour 4/6, 87036 Rende, Italy; 2 Environmental Chemistry Research Unit, Institute of Biology and Earth Sciences, Pomeranian University in Słupsk, 22a Arciszewskiego Str., 76-200 Słupsk, Poland; aleksander.astel@apsl.edu.pl; 3 Department of Science and Technology, University of Sannio, 82100 Benevento, Italy; domenico.cicchella@unisannio.it

**Keywords:** soil, potentially harmful elements, contamination, multidimensional spatial analysis, Calabria

## Abstract

The geochemical composition of bedrock is the key feature determining elemental concentrations in soil, followed by anthropogenic factors that have less impact. Concerning the latter, harmful effects on the trophic chain are increasingly affecting people living in and around urban areas. In the study area of the present survey, the municipalities of Cosenza and Rende (Calabria, southern Italy), topsoil were collected and analysed for 25 elements by inductively coupled plasma mass spectrometry (ICP-MS) in order to discriminate the different possible sources of elemental concentrations and define soil quality status. Statistical and geostatistical methods were applied to monitoring the concentrations of major oxides and minor elements, while the Self-Organizing Maps (SOM) algorithm was used for unsupervised grouping. Results show that seven clusters were identified—(I) Cr, Co, Fe, V, Ti, Al; (II) Ni, Na; (III) Y, Zr, Rb; (IV) Si, Mg, Ba; (V) Nb, Ce, La; (VI) Sr, P, Ca; (VII) As, Zn, Pb—according to soil elemental associations, which are controlled by chemical and mineralogical factors of the study area parent material and by soil-forming processes, but with some exceptions linked to anthropogenic input.

## 1. Introduction

Soil is a dynamic natural resource which, being the basic constituent of the trophic system, has a variety of vital functions for human and environmental life [1,2,3]. These functions are the result of the soil’s ability to control and maintain the materials and energy cycles between the atmosphere, groundwater and plant cover.

Many factors are responsible for the content, distribution and the behaviour of the chemical elements in soil, the first of which is the mineralogical and geochemical composition of the bedrock [4,5,6], followed by weathering [7,8] and soil formation processes (physical, chemical and biological), In addition, soils can be affected by the influence of phenomena such as the anthropogenic pollution [9,10,11,12,13,14] and the ratio and chemical composition of atmospheric depositions [15,16]. These latter sources of pollutants are widely distributed in urban soil, which is a repository of rainfall and wastewater discharge as well as atmospheric pollutants accumulated via deposition, Hence, the soil is an indicator of environmental contamination [17,18].

Differing to natural soils, which have a profile consisting of degrading vertical horizons, urban soils do not have a profile, and present great variability, both vertical and horizontal, because during their formation there are no pedogenetic processes, but instead the layering of debris, landfill, construction, and the remains of excavations of foundations [19,20]. Therefore, soils in the urban environment are the result of anthropogenic activities. Rapid industrialization and urbanization have occurred in most parts of the world during the last decades, and have stressed the soil with a growing pool of pollutants from different sources, posing a significant risk to humans and ecosystem [21,22]. The difference between soil pollution and air and water pollution lies in the fact that, in the first case, the pollutants remain for a long time in direct contact with the soil. Thus, the soil is continuously subject to pollution by toxic materials and dangerous micro-organisms which enter the air, water and the food chain [23,24]. Contact with contaminated soil may be also direct (inadvertent hand to mouth administration by children from using soil of parks and schools) or indirect (by inhaling soil contaminants which have vaporized) [25,26,27,28]. Longer contact with pollutants causes their accumulation in bones and organs. During this exposure, organ activities are disturbed, the nervous system is affected, and tumour diseases mature [29,30,31,32]. There are different types of environmental pollutants, and their potentially harmful elements (PHEs) are those that are particularly dangerous due to their ubiquity, toxicity, and persistence [33,34].

Therefore, evaluating soil pollution is of great concern. Due to urban soil spatial heterogeneity, a valuable approach to assess its quality is the application of multivariate statistics, since the environment is considered multivariate. According to many available recommendations [35], among the most effective data mining tools, those which enable unsupervised grouping based on mutual relationships between features of the analysed matrix, both of linear and nonlinear nature, are the most desired. One of the most powerful techniques for this purpose is use of the Self-Organizing Maps of Kohonen [36] because they enable display of the pattern present in multidimensional data sets on two-dimensional surface plots, are resistant against missing data and outliers, and their results are easily interpretable by decision-makers. In light of these issues, the aims of this study are (i) to individuate different types of pollution fonts controlling for a structure of monitoring data sets in a southern Italy area; (ii) to visualize geographical distribution of potentially harmful elements, and (iii) to identify high-risk areas that can be targeted for environmental risks and public health. These outcomes could be used by decision-makers working in the field of sustainable development implementation.

## 2. Materials and Methods

### 2.1. Study Area

**Figure 1:**
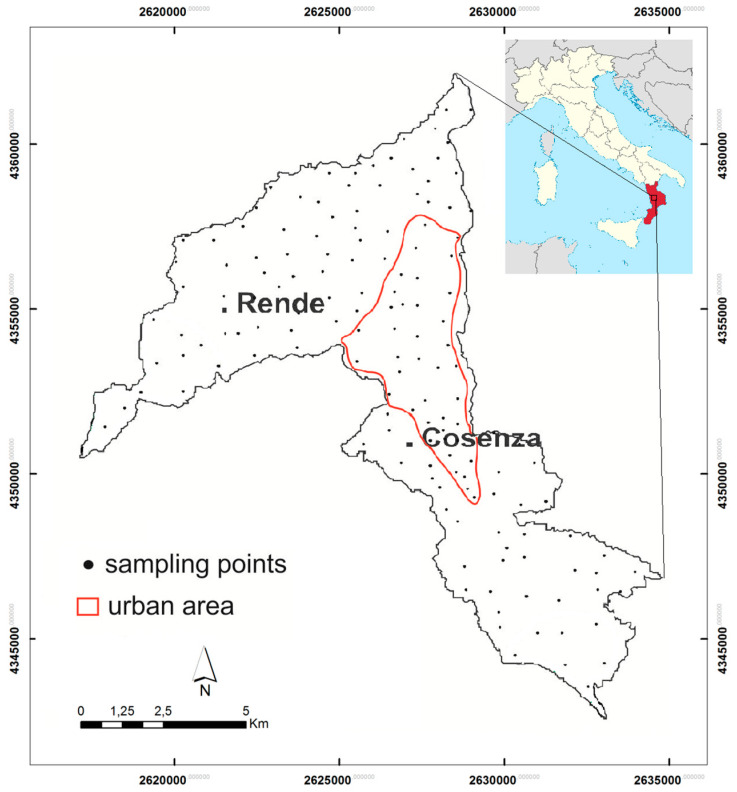
Study area with indication of sampling points and urban areas.

The study area is located in the NW sector of the Calabria region (southern Italy) inside the Crati graben and covers the Cosenza and Rende municipalities territory (Figure 1). Geologically, the study area represents a tectonic depression extending over 92 km^2^ bordered by NS, SW-NE and NW-SE-trending faults [37,38,39] associated with the horst-graben system of the Sila-Coastal Chain [40,41]. A tick succession of Pliocenic sediments made up of light brown and red sands and gravels, blue grey silty clays and silt interlayers, Pleistocene to Holocene alluvial sands and gravels and very small outcrops of Miocene carbonate rocks characterize the study area [42]. Sediments overlap a Palaeozoic intrusive-metamorphic complex formed by paragneiss, biotite schists, grey-phyllitic schists with quartz, chlorite and muscovite which, in some cases, are in a weathering process [43].

The soil map of the Calabria region at 1:250,000 scale [44], for the study area reports the presence of Fluvisols, Luvisols, Cambisols, Vertisols, Calcisols, Arenosols, Leptosols, Umbrisolsand Phaeozems. Properties, dynamics and functions of the studied soils are highly variable. For these, the average values are 17.59% for clay content, 56.50% for sand content, 6.84 for pH, 2.86% for organic matter, 0.25 µScm^−1^ for electrical conductivity, 16.14 meq 100 g^−1^ for CEC and 1.24 gcm^−3^ for bulk density.

Geomorphologically, a flat part including the urban area surrounded by hills, characterizes the study area. Falling inside the Mediterranean Sea, the Calabrian climate is typically Mediterranean, but the orography of the region affects it [45] with African warm air currents from its Ionian side and a western humid air current from the Tyrrhenian side.

The Cosenza-Rende area has a population of approximately 100,000 inhabitants and typical urban land use, such as housing and intense automobile traffic, with limited presence of industries, commercial activities, parks and gardens. For these characteristics, different potential sources of pollution can be recognized.

### 2.2. Soil Sampling and Analytical Methods

In this study, 149 soil samples were collected from residual and non-residual topsoil in gardens, parks, flowerbeds and agricultural fields (Figure 1) in the study area. In addition, two duplicate pairs were collected from every 10 sites and split in the laboratory to produce replicates. Before collecting samples, removal of the surface litter at the sampling spot was carried out. At each site, topsoil samples (0–10 cm depth from the surface) were collected from five locations at the corners and at the centre of a 20 × 20 m square with a hand auger and combined to form a bulked sample. Mixing of the samples thoroughly, and removal of foreign materials such as roots, stones, pebbles and gravel, were carried out. The final sample volume was 1–1.5 kg of material, reduced to about half by the following step of quartering. Sample preparation was started in laboratory by drying soil at 40 °C prior to analysis in order to obtain a water-free reference for elemental contents. Prior to further sample processing, the soil was adequately homogenized and then sieved to fine soil of ≤2 mm. Successive soil analyses were performed on fine soil, and analyte contents were based on fine soil as common reference for interstudy comparisons.

After appropriate preparation procedures, each soil sample was analysed by X-ray fluorescence spectrometry (XRF) for aluminium (Al), calcium (Ca), iron (Fe), potassium (K), magnesium (Mg), manganese (Mn), sodium (Na), phosphorous (P), silica (Si) and titanium (Ti), and by inductively coupled plasma mass spectrometry (ICP-MS) for arsenic (As), barium (Ba), cerium (Ce), cobalt (Co), chromium (Cr), lanthanum (La), niobium (Nb), nickel (Ni), lead (Pb), rubidium (Rb), strontium (Sr), vanadium (V), yttrium (Y), zinc (Zn) and zirconium (Zr).

Quality of the analysis was monitored by the simultaneous analysis of certified international reference materials AGV-1, BCR-1, BR, DR-N, GA, GSP-1, NIM-G, and analysis duplicates included in analytical procedure in the range of one in twenty in each batch. Errors of the estimate for the measured elements were determined by relative standard deviation (<5%) based on three replicates of one sample randomly chosen.

### 2.3. Data Processing Methods

Evaluation of the spatial distribution of pollutants is important to assess the anthropogenic burden on the environment. Numerous different chemometric approaches are available for multidimensional data mining; however, methods which can be used for unsupervised exploratory analysis and pattern recognition, as well as able to handle non-linear problems, are the most desired.

Among the different statistical tools applied, an increasing number of studies have used artificial neural networks to probe complex data sets, since the visual output of the SOM analysis provides a rapid and intuitive means to examine covariance between explanatory variables, especially when the relationships among them and phenomena under analysis are unknown, and possibly nonlinear. SOMs, while extensively used in many areas, have only recently been used in ecological applications [46]. Applications can be found in ecological community ordination and gradient analysis [47], and in characterization and prediction of water quality in rivers [48] and coastal areas [49]. Applications of SOMs in oceanography are quite recent, too, and consider mostly feature extractions from univariate data sets [50].

Self-organizing maps (SOMs), in particular, are a kind of unsupervised Artificial Neural Network (ANN) that have been becoming increasingly popular for the analysis of large multivariate data sets, since they provide a topology preserving nonlinear projection of the data set in a regular two-dimensional space, and therefore constitute a methodology for nonlinear ordination analysis.

The SOM technique, known as self-organizing maps of Kohonen, is able to deal with big data sets with the possibility of visually exploring the outcomes of the model in versatile 2D maps in which similar samples are mapped close together on a grid [51]. SOM is often used in association with other algorithms, such as K-means, Principal Component Analysis and Hierarchical Cluster Analysis, for further elaborating its outcomes. However, the majority of those associations are mainly methodological studies aimed at comparing outputs of various data mining strategies. Since the current research is a case-study, it was decided to use only the self-organizing map (SOM) algorithm, considering it one of the most current neural network architectures for exploratory data analysis, clustering, and data visualization.

Among the different statistical tools applied, an increasing number of studies have used artificial neural networks to probe complex data sets, since the visual output of the SOM analysis provides a rapid and intuitive means to examine covariance between variables.

SOM is a kind of artificial neural network performing a non-linear projection of the original data space onto a two-dimensional space of neurons. It consists of two layers: the first represents input nodes (one per variable) connected to the samples, while the second one (an output layer) is a set of neurons organized on an array. A preliminary number of neurons can be determined according to one of the most accepted recommendations where *n* = (number of samples)^(−1/2) [52], while the final map dimension ratio is usually slightly modified based on analysis of topographic and quantization errors (TE and QE, respectively). In general, a matrix of input vectors representing the variability and relationships of the experimental data is initialized by a series of parameters (i.e., shape of the map, shape of the map units, number of map neurons, map initialization matrix, distance function, neighbourhood function, number of epochs, etc.) retaining the number of variables of the experimental data. This input matrix becomes “the map”, usually represented in a two-dimensional plot where the map vectors are called prototypes (or neurons). Then, each vector of the experimental data is presented to the algorithm, and it finds the prototype most similar to the experimental vector and adjusts it together with all surrounding prototypes to be even more similar to the experimental vector. When all the experimental vectors are presented to the algorithm, a single iteration is finished; usually, several iterations are needed to convergence. In the current study, the following initializing parameters were used: rectangular shape of the map, hexagonal shape of the map unit, 66 map units, random initialization, Euclidean distance to find the best prototype and adjust the surrounding neurons, and Gaussian neighbourhood function to establish how the neurons around the best prototype are updated during the training process. Once the SOM has converged, the weight vectors of the elements are fed into a non-hierarchical K-means algorithm to extract the neurons of the best similarity. Separating by K-means requires the user to decide the final number of k clusters the algorithm is converged into. Diverse values of k (predefined number of clusters) were tested and the sum of square for each run was calculated. Lastly, the best classification with the lowest Davies-Bouldin index (D-B) was selected. D-B index is a function of the ratio of the sum of within-cluster scatter and between-cluster separation [53]. The non-parametric Kruskal-Wallis test was performed to evaluate the significance of the cluster pattern.

All calculations in this study were performed by applying Matlab 2020 (Mathworks, Inc., Natick, MA, USA) and TIBCO Statistica 13.0 (TIBCO Software, Palo Alto, CA, USA) running on a Windows 10 platform. 

## 3. Results and Discussion

Table 1 presents the descriptive statistics for the soil data. Except for Na and Si, a positive skewness is observed for all elements (Table 1), and a kurtosis which ranges from slight (0.04) to high (46.74).

To analyse the spatial variations of elemental concentrations, the data set, consisting of analytical results from urban and peri-urban soil samples, was arranged in a two-way array of 25 variables, and the SOM algorithm was deployed. Apart from the methodological information presented in the section above, the detailed theoretical background of the SOM approach can be found elsewhere [54,55,56,57]; however, it is worth mentioning that here the SOM was successfully applied in assessment of soil pollution with PHEs [58], and heavy metals [59,60,61,62,63] as well as PCDD and PCDFs [64]. In Tao et al. [58] the distribution of PHEs in surface soil was examined. Yotova et al. [59] focused on toxic elements present in soil and their phytoavailability in an industrial area with copper mining factories and a smelter. In Yang et al. [60], soil samples were collected in several sites in a vast Chinese region and analysed for toxic elements presence. Kosiba et al. [61] compared the use of SOM with three other statistical techniques for assessing soil quality in a Polish area and its impact on the diffusion of a pathogen on a specific plant species. Dai et al. [64] evaluated the dioxin content in soil at different depths and in different years in a river floodplain, while in Nadal et al. [62] the use of the SOM allowed identification sites differently impacted by heavy metal pollutants in a petrochemical industrial area. Cheng et al. [63] proposed a SOM model built from a dataset composed of toxic metal content of soil and sediment samples collected at different depths from cascading reservoir catchments of a Chinese river. Having in mind the facts mentioned above, in the present study, exploratory data analysis, clustering and data imagining were approached by the self-organizing map (SOM) algorithm, which represents a powerful neural network architecture for these topics.

**Figure 2:**
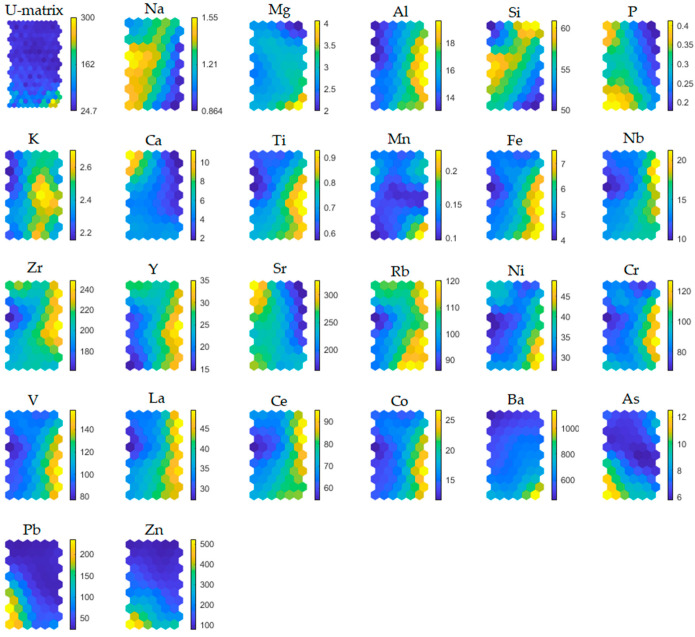
Component planes for all sampling sites and parameters. U-matrix visualizes distances between neighbouring map units and helps to identify the cluster structure of the map. High values of the U-matrix indicate a cluster border, uniform areas of low values indicate clusters themselves; each component plane shows the values of one variable in each map unit. Both grey-tone pattern and grey-tone bar labelled as “d” deliver information regarding compounds/element abundance calculated through the SOM learning process.

According to one of the most accepted recommendations [52], the total number of Kohonen’s map neurons was estimated as *n* = 5 * (149)^(−1/2) ≈ 61. Since there was more than one possible combination of the final dimension which was close to the dimension obtained by Vesanto’s formula (i.e., 10 × 6, 8 × 7, 9 × 7, 11 × 6), quantization (QE) and topographic errors (TE) were calculated in all cases. Finally, the chosen dimensionality of the 11 × 6 had the lowest values of errors (QE = 0.231, TE = 0.011). Once the SOM’s grid has been optimized, the U-matrix and the individual variable planes based on hexagonal lattice were visualized (Figure 2).

A component plane, scaled to represent the range of changeability of a parameter, is associated to each variable while the corresponding hexagon (i.e., top-left one of coordinates row × column = 1 × 1) of the consecutive plane represents the changeability of the given parameters for the same set of samples. Based on this, the component planes can be used to visualize possible correlation among the variables, while the U-matrix can be used to identify the possible presence of different clusters of data. By the analysis of planes, high concentration values of Al, Ti, Fe, Y, Rb, Cr, V, La and Ce, which are generally located in the top of the planes, and the highest concentration values of Pb and Zn in the bottom-left part of the planes, were observed.

Since PHE concentration in soils depends both on the nature of bedrock, on abiotic and biotic factors, and human activities, accurately extracting key features and characteristic patterns of variability from an elemental large data set is essential to correctly determining the sources. For this, the relationship between elements in the soil matrix gives information on PHE sources and pathways in the geo-environment. In fact, positive correlations between elements, inspected by comparing component planes, suggest that pairs in the soil samples are from the same source. Conversely, negative correlations suggest different origins between the element’s pairs which, therefore, can be considered unrelated to their geochemical dynamics.

Scaling the weights vectors of each plane in the range between 0 (the least positive) and 1 (the most positive), the set of variables could be separated into several groups of similarity representing their mutual directly or inversely proportional correlations.

- Cr, Co, Fe, V, Ti, and Al with clear consistent patterns of the highest weights in the top-left part of the planes and the lowest weights in the middle-bottom section of the planes. These variables are all positively correlated and probably not associated with anthropogenic sources, but supposedly related to the predominant rock-forming elements constituting the soil parental materials. Indeed, the higher values of this group’s element concentrations were located mostly in the NW and SE sectors of the study area where a very low road network density occurs and where there is the occurrence of ultrabasic rocks, found below the Pliocene deposits, in which these elements are predominant. The igneous-metamorphic complex can be ascribed to the pile of tectonic nappes forming the mountain chain of the northern Calabrian Arc, described in [65], which includes an intermediate structural element made up of ophiolite-bearing units [66] that mostly extend along the Tyrrhenian side of the arc to form a westward convex arc-shaped belt separated from the southern Apennines by the roughly E-W trending left-lateral strike-slip fault zone. This unit is represented by a tectonic mélange constituted by a monotonous sequence of phyllites, quartzites, and calcschists, including metric to kilometric lens-shaped blocks of ophiolitic rocks. These rocks are mainly constituted by serpentinized ultramafics, and by glaucophane-bearing meta-basites, with remnants of their sedimentary cover and rare meta-gabbros [65]. In particular, the geochemical behaviour of V resembles that of Fe which can substitute in Fe-Mg silicates (amphiboles, pyroxenes, micas). This elemental association confirms that the soils are controlled by the same typically lithogenic elements associated with silicate minerals.
- Ni and Na with clear opposite patterns of the highest and the lowest weights of Ni and Na occur, respectively, in the top-left triangle of hexagons. By contrast, the lowest and the highest weights of Ni and Na, respectively, cover the bottom-right triangle of hexagons. One important observation that arises from the calculation of the correlations for these elements is that Ni does not have any positive relation with Na. The absence of this correlation could be attributed to the influence of the distribution of these elements by anthropogenic activities.
- Y, Zr and Rb with consistently increasing weights occur in the top-half part of the planes and descending weights in the bottom-half. Such a pattern indicates that Y, Zr and Rb are positively correlated and considered to indicate provenance compositions as a consequence of their immobile behaviour [67]. Zr is enriched in silica rich sediments compared to the associated shales, which suggests its propensity to be preferentially concentrated in coarser sediments. Many soil samples can be attributed to the compositional field in which the local content of marbles, sandstones, and gneisses are part, indicating a strong lithological influence on element concentrations. Therefore, Y, Zr and Rb association prove to be of undoubted geogenic origin.
- Si, Mg and Ba have patterns that, in general, are similar to the patterns observed in the case of Ni and Na. The highest weights for Mg and Ba are observed only for single hexagons of coordinates 1 × 1, 1 × 2 and 2 × 1, while for those hexagons, relatively low weights of Si occur. By contrast, in the bottom-right triangle of hexagons, high weights for Si correspond with low weights for Mg and Ba. Generally, such patterns indicate that Si is negatively correlated with Mg and Ba, while Ba is positively correlated with Mg. Ba is a trace element common in alkali feldspars and biotite. The lack of a clear correlation between Al, Rb and Sr and Ba indicates a relationship between Ba and mica components, or that Ba was lost at an early stage in weathering of feldspars.
- Nb, Ce and La, with the highest values of weights, occur in only a few hexagons in the top-right triangle of the planes. Nb, Ce and La, belonging to the rare earth elements (REEs), show positive correlations, explaining their similar behaviour in soil samples. Their primary source is accessory minerals in magmatic rocks, e.g., monazite, xenotime and allanite. This could explain their common geogenic sources.
- Sr, P and Ca, with compatibly the highest weights, occur in a thinly vertical belt of hexagons located on the left-hand side of the planes. Such a pattern indicates a strong positive correlation between Sr, P and Ca, which confirms a mineralogical common source of elemental association. This may be due to Sr geochemical affinity with Ca [68]. Sr is a relatively common element that substitutes for Ca in crystal lattices of rock-forming minerals, including feldspars and plagioclase, as in the study area.
- As, Zn, Pb, with compatibly the highest weights. occur in only a few hexagons located in the bottom-left triangle of the planes. The consistent colour indicates that As, Zn and Pb have a strong positive correlation, and their concentrations are higher in soil next to roads than in the soils away from them. This indicates that larger concentrations of these elements are related to road traffic. Consequently, their positive correlation allows us to draw conclusions about their common source linked to anthropogenic activities conducted in urban environments. These elements are, indeed, present in vehicle fuel, being used for increasing gasoline antiknock.

**Figure 3:**
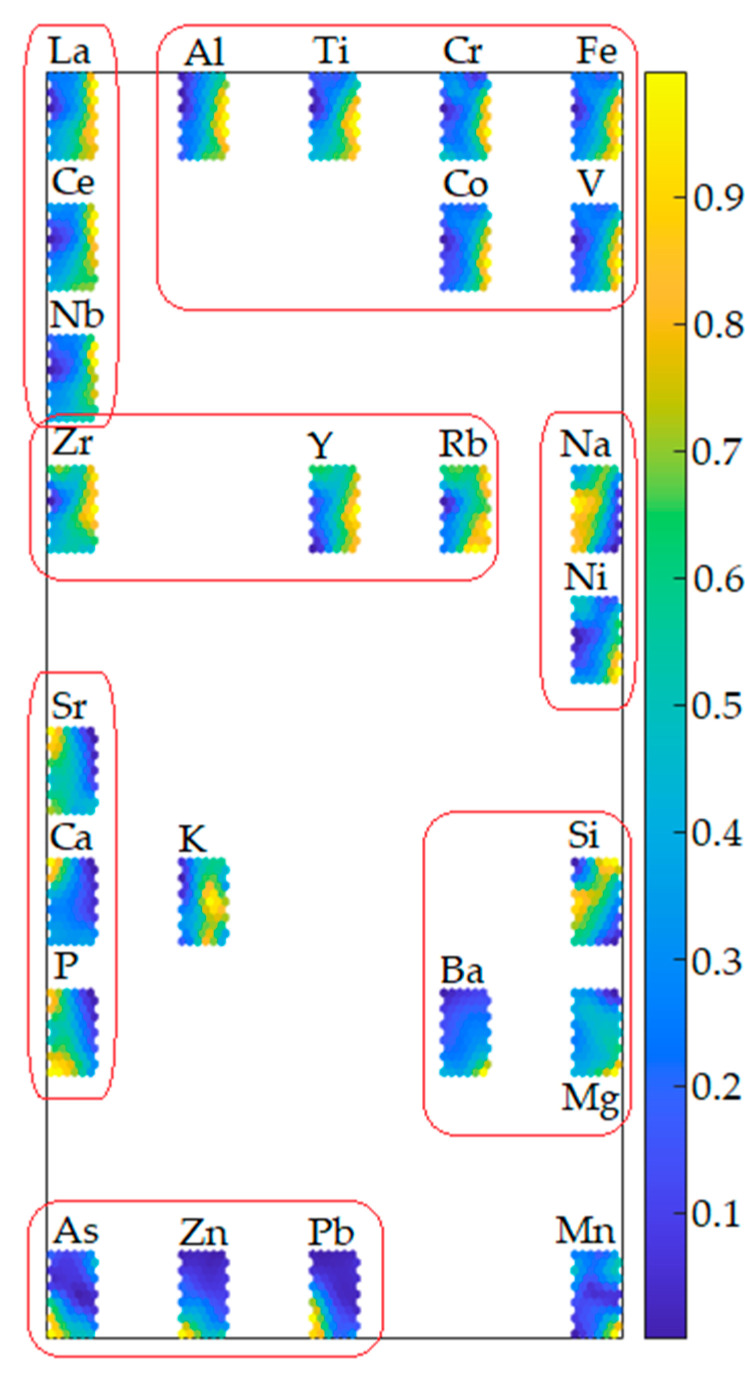
Soil quality parameter similarity pattern obtained by self-organizing mapping. An analysis of the distance between variables on the map connected with an assessment of the colour-tone patterns provides semi-quantitative information about the nature of correlations between them.

The set of component planes, with weights scaled in the range 0–1 grouped according to their correlations, is presented in Figure 3.

**Figure 4:**
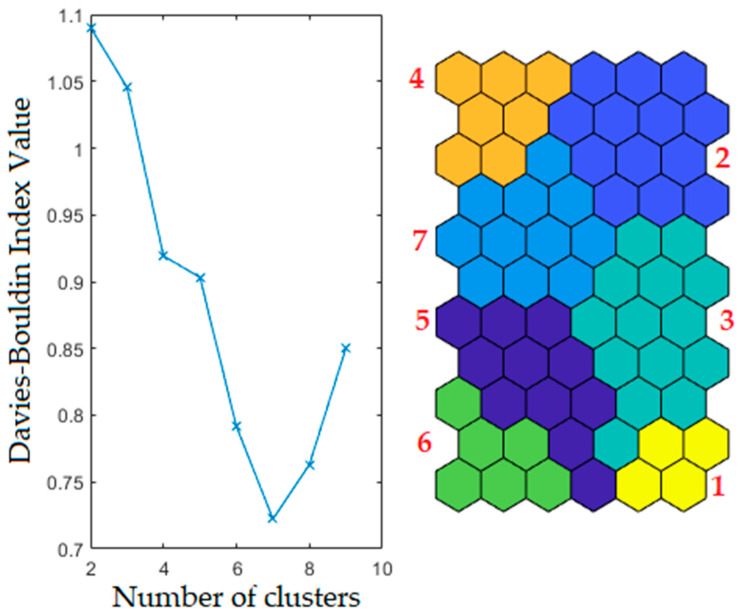
Clustering patterns according to the Davies-Bouldin index minimum value.

The significant information deriving from the SOM theory, that each node of the SOM map could be consecutively referred to one or more samples, leads to the conclusion that the differentiated structure of PHEs abundance (reflected in different colour scales in the planes) revealed the presence of numerous similarity clusters in the set of samples. Consequently, weight vectors of the converged map were clustered based on a K-means clustering mode. Some predefined numbers of clusters were tested, and the sum of squares for each run was calculated. The best partition was gained for a seven-cluster configuration having the lowest Davies-Bouldin index value (Figure 4).

**Figure 5:**
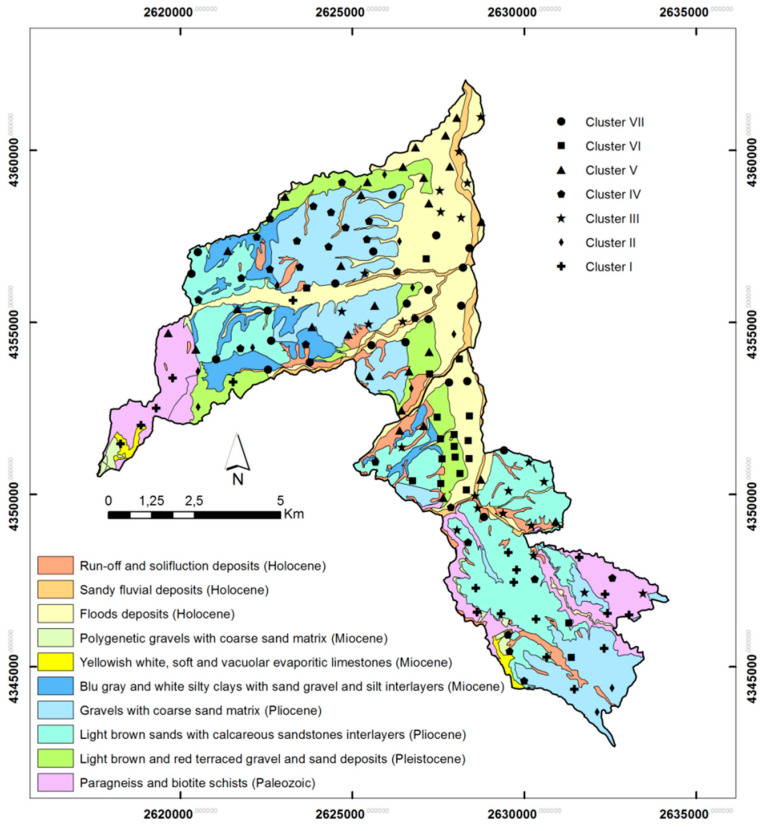
Geological setting of the study area and localization of sampling according to cluster classification. The seven identified clusters—(I) Cr, Co, Fe, V, Ti, Al; (II) Ni, Na; (III) Y, Zr, Rb; (IV) Si, Mg, Ba; (V) Nb, Ce, La; (VI) Sr, P, Ca; (VII) As, Zn, Pb—representing the seven groups in which the elements are associated according to local lithologies, are indicated.

**Figure 6:**
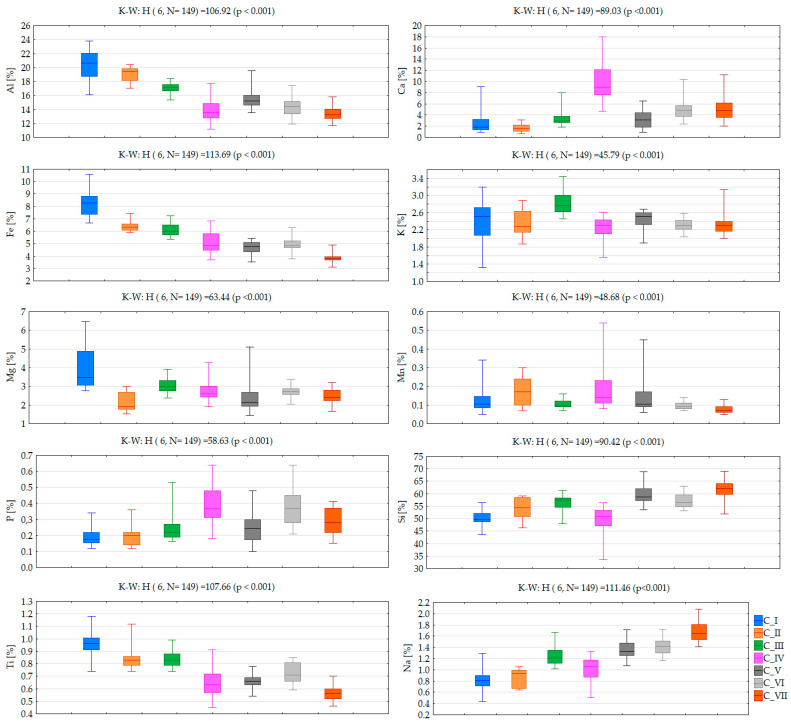
Compound concentration values according to clustering patterns (central line: median, box: 25–75% percentile, whiskers: minimum-maximum) with statistical assessment of differences between clusters based on the Kruskall-Wallis non-parametric test (K-W).

**Figure 7:**
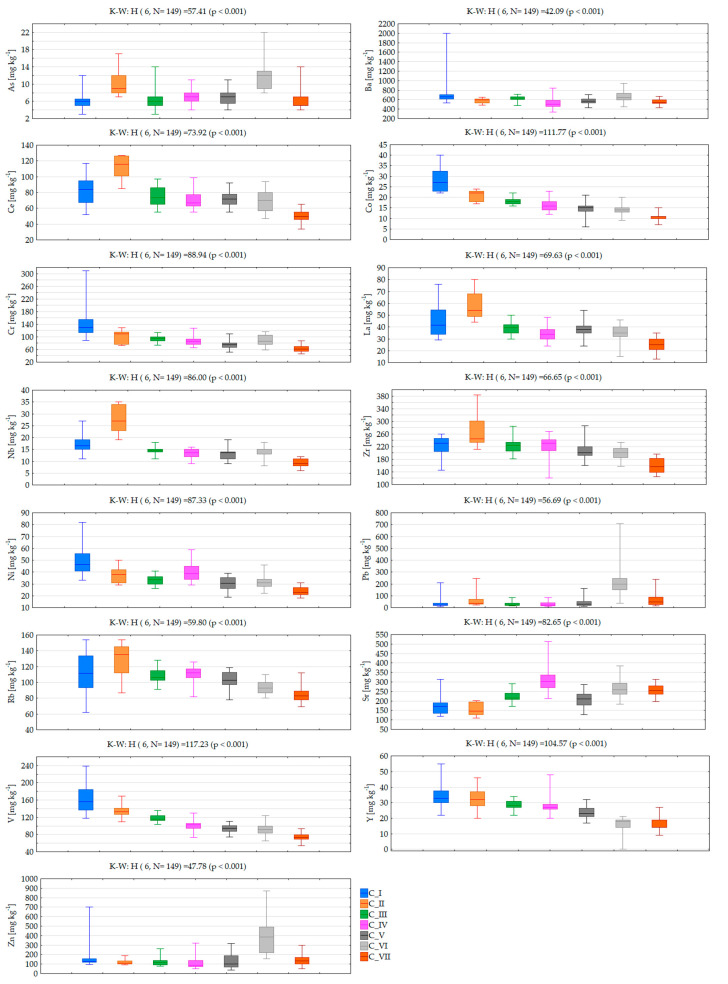
PHE concentration values according to clustering patterns (central line: median, box: 25–75% percentile, whiskers: minimum-maximum) with statistical assessment of differences between clusters based on the Kruskall-Wallis non-parametric test (K-W).

According to SOM theory, the node (map neuron) with a weight vector closest to the input sample vector is identified as the best matching unit, and the number of tagging is summarized. Lastly, the distribution of the sample vectors along a Kohonen map can be analysed by decoding the best matching unit selection events. Clusters I-VII (consecutively named as C_I-C_VII) include numerous numbers of 149 soil samples (C_I-20, C_II-11, C_III-22, C_IV-26, C_V-28, C_VI-19, C_VII-23). Cluster distribution of investigated soil samples in the study area according to the local geological setting is presented in Figure 5. Comparison of initially determined PHEs concentrations in soil samples with the clustering results allowed for the assignment of clustering patterns to factors impacting soil quality. Comparison of analyte concentration values according to clustering pattern is presented in Figure 6 (concentration at % level) and Figure 7 (concentration at mg kg^−1^ level) together with a statistical assessment from the non-parametric Kruskal-Wallis test. 

Among the seven clusters, C_I includes 20 samples (13.4%) with the highest concentration of Mg, Al, Ti, Ni, Fe, Y, Cr, V, Co and Ba. Most of the samples included in C_I was collected in peri-urban soils and in areas in which Paleozoic paragneiss and biotite schists occur. More precisely, the observed association clustered in C_I can be clarified considering the presence in the study area of ultrabasic rocks in which these elements are principal. Highest baseline concentrations of these elements seem to be highly associated with the igneous-metamorphic complex found below the Pliocene deposits that outcrop mostly in the NW and SE sectors of the territory. This structure represents the pile of tectonic nappes forming the mountain chain of the northern Calabrian Arc and contains an intermediate structural element made up of ophiolite-bearing units.

C_II consists of only 11 (7.4%) samples collected in peri-urban soils. These samples were characterized by the lowest concentration of Mg and Ca, with the highest abundance of REEs such as La, Ce, Nb and Zr. Such a phenomenon indicates that REE content is associated with alkaline igneous rocks and carbonatites, which are igneous rocks derived from carbonate-rich magma rather than silica-rich magma [69].

C_III includes 22 samples (14.8%) with the highest concentration of K and relatively low abundance of Zn, Mn, Ni, Ba, Pb, As. The majority of these samples were collected in soils along the part of the Crati river falling in the study area, and their composition indicates the presence of organic matter, suggesting that this might play a role in increasing K adsorption rate. As can be seen, samples clustered in C_I-C-III as a set, in comparison to the rest of clusters, were characterized by higher concentrations of Al, K, Ti, and Fe, with lower concentration of P and Sr. Moreover, samples from C_I and C_II were characterized by the highest concentration range for REEs. The content of REE in soil, without other inputs, is influenced by the parent material and on geochemical processes such as mineral weathering, which is an important input of elements into the soils [70]. Twenty-six samples clustered in C_IV (17.4%) were, in general, grouped together based on the lowest content of Al and Si, relative to minimal concentrations among of the other samples, and the highest concentration of P, Ca, Sr. According to their location, this suggests that the underlying rocks are the major source of P. C_V, consisting of 28 (18.8%) samples, represents soils with moderate concentrations of the majority of investigated elements. It seems they are clustered separately due to a relatively large range of determined concentrations for Mg and Mn. C_VI includes 19 (12.7%) soil samples in which their chemical composition is dominated by relatively high concentration of P, Zn, and Sr. These samples were additionally characterized by the highest concentration and range of values for Pb and As, and lowest the abundance of Y. The soil samples characterized by these elemental contents are distributed in the urban area, where road networks and vehicular traffic are intense, and, consequently, higher Pb contents occur. Particularly, soils close to high traffic roads of the study area showed the highest Pb and Zn baseline values. These elements are included in vehicle fuel for increasing gasoline antiknock. The last C_VII includes 23 samples characterized by the lowest concentration of the majority of elements, such as Ti, Mn, Rb, Ni, Fe, Zr, Y, Cr, V, La, Ce and Co. In general samples clustered in C_V-C_VII show consistent chemical composition with the exception of some elements, determining their separation in a single cluster. As can be seen in Figure 6, a monotonic increasing trend of determined concentration values from C_I to C_VII is observed for Si, Na, and Sr, while much more frequently observed was a decreasing trend for Al, Ti, Rb, Ni, Fe, Y, Cr, V and Co.

## 4. Conclusions

Correct monitoring and management of potentially harmful elements are key issues for urban and peri-urban soil knowledge, linking PHE concentrations at sites in which geogenic or anthropogenic input occur. In this study, evaluation of the usefulness of a powerful approach, such the SOM algorithm, for multidimensional geochemical data analysis and modelling problems of environmental pollution, was performed using data sets obtained by comprehensive monitoring of PHE content in the municipalities of the Cosenza-Rende area (Calabria, southern Italy). In the study area, a total of 149 soil samples, collected in residual and non-residual areas, parks, flowerbeds and agricultural fields, were investigated for 25 elements in order to better understand influences on soil geochemistry.

A self-organizing map (SOM) was selected as a powerful approach in soil science application for spatial distribution and geochemical mapping. A combination of the analysis of major metals, minor metals and PHEs, with the statistical treatment of SOMs, showed the geolithological formations and anthropogenic pressure on the territory. The association between the neurons and variables achieved by an unsupervised procedure performed by the SOM technique, allows recognition of high-risk areas which can represent environmental hazards and public health risks. By using the SOM method, the occurrence of anomalies ascribable to anthropogenic input in urban soils, referring to elements such as Pb and Zn, and of some geogenic anomalous high values of As, Cr, and V mainly identified in peri-urban areas, was recognized. The SOM was employed to cluster the data, and results presented a classification in seven clusters—(I) Cr, Co, Fe, V, Ti, Al; (II) Ni, Na; (III) Y, Zr, Rb; (IV) Si, Mg, Ba; (V) Nb, Ce, La; (VI) Sr, P, Ca; (VII) As, Zn, Pb—mainly determined by the chemical and mineralogical factors typical of the geological setting of the study area, and by soil forming and weathering processes. Among them, C_II and C_VII can be linked to anthropogenic input. However, in general, more contamination was identified in urban soils than in peri-urban ones.

In summary, the main outcomes of the study are as follows:

1. SOM was verified as a promising approach for pattern recognition and, in particular, for delineating pollution patterns of soil;
2. the main factors that influence PHE concentration in the Cosenza-Rende area were associated with geological setting and human activities;
3. classification of soil patterns provides a great deal of information enhancing risk status source identification, which can be used for decision making.

The paper contains an important methodological novelty. In fact, it proposes the application of an existing methodology for data analysis to a new class of problems. Its results can have a valuable role in identifying polluted areas and proposing remedial action aimed at reducing health risks to people. Further development of this tool should also help soil scientists to identify novel relationships about already studied phenomena, and act as a hypothesis generator for traditional research, as well as supplying clear and intuitive visualization of the environmental phenomena studied.

## Figures and Tables

**Table 1:** Basic statistics for soil samples.

	Unit	Min	Max	Mean	Median	Lower Quartile	Upper Quartile	S.D.	Skewness	Kurtosis
**Al_2_O_3_**	%	11.19	23.79	15.89	15.38	13.65	17.48	2.80	0.69	0.04
**CaO**	%	0.66	17.97	4.76	3.84	2.39	6.19	3.39	1.56	3.27
**Fe_2_O_3_**	%	3.11	10.58	5.47	5.13	4.42	6.16	1.47	1.03	1.01
**K_2_O**	%	1.32	3.45	2.41	2.40	2.22	2.60	0.34	0.06	1.28
**MgO**	%	1.45	6.47	2.81	2.74	2.32	3.06	0.81	1.45	3.27
**MnO**	%	0.05	0.54	0.13	0.10	0.09	0.14	0.08	2.67	8.4
**Na_2_O**	%	0.44	2.08	1.23	1.23	1.02	1.46	0.34	−0.03	0.31
**P_2_O_5_**	%	0.10	0.64	0.29	0.26	0.19	0.36	0.12	1.00	0.72
**SiO_2_**	%	33.45	68.98	55.72	56.31	51.98	59.46	5.89	−0.52	0.87
**TiO_2_**	%	0.45	1.18	0.73	0.71	0.61	0.83	0.15	0.47	0.2
**As**	mg kg^−1^	3	22	7	7	5	9	3	2	3.47
**Ba**	mg kg^−1^	335	2000	603	592	530	643	153	5	46.74
**Ce**	mg kg^−1^	34	127	73	70	60	82	19	1	0.57
**Co**	mg kg^−1^	6	40	17	16	13	20	6	1	2.02
**Cr**	mg kg^−1^	46	309	91	86	73	103	32	3	15.44
**La**	mg kg^−1^	13	80	38	37	31	42	11	1	1.91
**Nb**	mg kg^−1^	6	35	14	14	11	15	5	2	3.87
**Ni**	mg kg^−1^	18	82	35	33	28	40	10	1	2.95
**Pb**	mg kg^−1^	8	708	64	31	20	69	85	4	22.56
**Rb**	mg kg^−1^	62	154	105	105	92	114	18	0	0.2
**Sr**	mg kg^−1^	109	514	234	233	194	271	64	1	1.66
**V**	mg kg^−1^	54	239	107	102	87	123	31	1	2.46
**Y**	mg kg^−1^	0	55	25	26	19	30	8	0	1.08
**Zn**	mg kg^−1^	38	871	167	127	93	189	131	3	8.7
**Zr**	mg kg^−1^	121	383	209	209	186	233	41	0	1.74

## Data Availability

Not applicable.
